# Establishment of Cephalic Index Using Cranial Parameters by Computed Tomography in a Sampled North Indian Population

**DOI:** 10.7759/cureus.15421

**Published:** 2021-06-03

**Authors:** Sachin Khanduri, Saif Malik, Nazia Khan, Yunus D Patel, Asif Khan, Harleen Chawla, Vishwesh Singh, Ashok Gupta, Juned Shaikh, Saim Siddiqui

**Affiliations:** 1 Radiology, Era's Lucknow Medical College and Hospital, Lucknow, IND; 2 Radiodiagnosis, Era's Lucknow Medical College and Hospital, Lucknow, IND; 3 Radiodiagnosis, Era’s Lucknow Medical College and Hospital, Lucknow, IND

**Keywords:** anthropometry, cranial index, computed tomography, cephalic index, max cranial breadth of heads, max cranial length of heads, mesocephalic, dolicocephalic

## Abstract

Background: Cephalic index (CI) also called cranial index is the ratio of maximum breadth to a maximum length of head. The purpose of the study was to study anthropometry of cranial parameters using the computed tomography (CT) scans to establish the CI of the sampled population in North India.

Materials and methods: The cross-sectional study was carried on the subjects of age group ranging from 6 to 95 years of either sex (total 1000 subjects; 540 male and 460 female) in the radio-diagnosis department of Era’s Medical College Lucknow, UP, India. The measurement of maximum cranial breadth (MCB) and maximum cranial length (MCL) were taken on a CT scan machine and recorded for analysis. When associating the measures of precision for different subgroups, a one-way analysis of variance (ANOVA) was used for modest and efficient errors. Multivariate logistic regression analysis was used to identify factors affecting the CI estimation like age, interzygomatic length (IZL), orbital length (OL), MCB, and MCL.

Result: Out of 1000 studied subjects, the majority 234 (23.4%) of the subjects belong to the 21-30 years age group. MCB of heads and MCL of heads in different ages and on applying the one-way ANOVA association was statistically significant and CI was statistically insignificant. Pearson correlation between the CI and other parameters like age, MCB of heads, and MCL of heads, and a statistically significant correlation was seen with each other. Dolichocephalic types of the skull are found more in male subjects, and brachycephalic type of skull is more common in female subjects.

Conclusion: The average CI of our study was 76.67±3.18. This shows that northern India's dominant head shape, especially in the Lucknow region, was dolichocephaly. Thus, the CT scan is proven an essential modality in the assessment of cranial parameters in anthropometry.

## Introduction

Cephalic index (CI), also known as the index of breadth or cranial index, is one of the major parameters that help to distinguish between the different races of humans. The CI was described by a Swedish professor (1796-1860) and was first used in physical anthropology to categorize remains of ancient humans found in Europe [1].

The dimensions of the human body are affected by biological, ecological, geographical, sex, racial, and age factors [2]. The CI is measured as the breadth of the skull multiplied by 100 and divided by length. CI is classified into three broad categories: dolichocephalic (less than 75), mesaticephalic (75 to 80), and brachycephalic (greater than 80). Australian and native Southern Africans are dolichocephalics, Chinese and European skulls are mesaticephalic, and Andaman Islanders and Mongolians have brachycephalic skulls [3,4].

Currently, CI is commonly used to portray individuals' appearances and for roughly calculating the age of the fetuses for obstetrical and legal reasons. So it can be widely used in various investigations in forensic labs. Comparison of changes in CI among offspring, parents, and siblings may give a hint to the genetic transmission of the inherited characters. CI is the jargon used in anthropology for having an easy identifying module or the numerical to differentiate the given individual, either into sex or race or even as the identity of the individual [5].

Cephalometric examination relies upon the cephalometric radiography to examine connections in the middle of delicate and hard tissue points of interest and may be utilized to investigate facial development differences from norm preceding treatment, amidst treatment to evaluate progress or at the end of treatment to determine that the objectives of the treatment have been met [6]. The lateral cephalometric radiograph is a radiograph of the head brought with an X-ray beam opposite to the patient's sagittal plane [6,7]. The regular position of the head is a systematized introduction of the head which is reproducible for each person and is used as a process for examination of the dentofacial morphology.

More recently, computed tomography (CT) has permitted complete imaging of the whole craniofacial complex. This technology is enhanced further by the computer software that allows three-dimensional (3D) reconstructions of the CT slices, allowing the life-like appearance of the face and skull for measuring purposes. CT has provided the latest tools for medical investigation and has been used widely for pre and post-operative imaging when assessing patients with craniofacial abnormalities [8].

Regarding the effect of ethnic, racial, and geographical factors on the head dimensions, the present study was performed to find the CI of the adult population, to determine the dominant head type and sexual dimorphism in Northern Indian adults populations of age group >18 years of Lucknow. The observations and findings of the present study will provide a platform for similar cephalometric studies done on various communities, castes, races of particular geographic zones.

## Materials and methods

The present cross-sectional study was performed in the Department of Radio-Diagnosis, Era’s Medical College, Lucknow, UP, India. One thousand subjects of different age groups from 6 to 95 years of both sexes were selected. The subjects chosen were apparently healthy and without any visible spinal or cranial deformity. Any subject with congenital or acquired cranial, spinal, or bone deformity, i.e., scoliosis, kyphosis, etc., were excluded. The CT scans for facial bones were performed on a 384 slice Dual Energy CT scanner (Somaton Force, Seimens Healthcare, Erlangen, Germany) and all images were post-processed on a workstation using Synovia software (Synovia Solutions, Fort Worth, TX) that allowed analysis of images using three material decompositions. Examinations were evaluated by an experienced radiologist.

The formula used to calculate the CI:

CI = [Maximum Cranial Breadth (MCB)/Maximum Cranial Length (MCL)] * 100

On the basis of the CI, skulls were classified as “According to Modi’s Medical Jurisprudence and Toxicology, the skulls having CI between 70 and 74.9 are called dolichocephalic or long headed" [9,10]. This type is common among Aborigines and pure Aryans. Skulls having CI from 75-79.9 are grouped under mesocephalic and are common features of Europeans and Chinese. The more CI from 80 to 84.9 is called brachycephalic or short-headed. The Mongolian race is an example of brachycephalic heads [11].

Statistical analysis

Statistical analysis was done by using the Statistical Package for Social Sciences (SPSS-23, IBM Corp., Armonk, NY) program. Data were expressed in the form of frequency in percentage and value in mean and standard deviation. When associating the measures of precision for different subgroups, a one-way analysis of variance (ANOVA) was used for the modest and efficient errors. Multivariate logistic regression analysis was used to identify factors affecting the CI estimation like age, interzygomatic line (IZL), orbital length (OL), maximum breadth of heads, and head’s maximum length. A p-value of <0.05 was used as the criterion of statistical significance.

## Results

Out of the total 1000 studied subjects, the majority 234 (23.4%) of subjects belonged to the age group 21-30 years and the mean age of the total studied subjects was 43.43±20.71 years (range 6-95 years; Table 1).

**Table 1:** Age and sex distribution of the studied cases

Age group (years)	Sex		Total
	Male (n=540)	Female (n=460)	
≤20	74 (13.7%)	68 (14.8%)	142 (14.2%)
21-30	130 (24.1%)	104 (22.6%)	234 (23.4%)
31-40	60 (11.1%)	72 (15.7%)	132 (13.2%)
41-50	56 (10.4%)	60 (13.0%)	116 (11.6%)
51-60	64 (11.9%)	56 (12.2%)	120 (12.0%)
61-70	80 (14.8%)	60 (13.0%)	140 (14.0%)
71-80	64 (11.9%)	32 (7.0%)	96 (9.6%)
>80	12 (2.2%)	8 (1.7%)	20 (2.0%)
Mean age (mean±SD) years	44.37±21.16	42.32±20.16	43.43±20.71

Male subjects (54.0%) were dominant over females subjects (46.0%; Table 1). The MCL of subjects was 17.50±0.57 mm, mean MCB of heads was 13.40±0.39 mm, mean IZL was 12.17±0.49 mm, OL was 4.10±0.19, and mean CI was 76.67±3.18 (Table 2).

**Table 2:** Distribution of maximum cranial length, maximum cranial breadth, interzygomatic line, orbital length, and cephalic index in both sex groups

Variables	Total (n=1000)	Sex		P-value
		Male (n=540)	Female (n=460)	
Maximum cranial length (mm)	17.50±0.57	17.69±0.46	17.28±0.60	<0.001
Maximum cranial breadth (mm)	13.40±0.39	13.36±0.38	13.45±0.38	<0.001
Interzygomatic length (mm)	12.17±0.49	12.34±0.47	11.97±0.42	<0.001
Orbital length (mm)	4.10±0.19	4.16±0.19	4.04±0.17	<0.001
Cephalic index	76.67±3.18	75.59±2.64	77.94±3.30	<0.001

The distribution of MCL, mean MCB, mean IZL, OL, and mean CI in different gestational ages was statistically significant (p<0.05; Table 3).

**Table 3:** Distribution of maximum cranial length, maximum cranial breadth, interzygomatic length, orbital length, and cephalic index in different age groups

Age group (years)	N	MCL	MCB	IZL	OL	CI
≤20	142	17.59±0.45	13.40±0.33	12.21±0.51	4.08±0.16	76.27±2.97
21-30	234	17.50±0.57	13.50±0.37	12.19±0.47	4.11±0.19	77.25±3.18
31-40	132	17.62±0.44	13.39±0.35	12.04±0.49	4.09±0.16	76.10±3.34
41-50	116	17.45±0.46	13.39±0.35	12.13±0.48	4.10±0.19	76.79±2.99
51-60	120	17.51±0.66	13.42±0.27	12.02±0.46	4.04±0.17	76.73±3.15
61-70	140	17.43±0.68	13.30±0.34	12.29±0.46	4.16±0.21	76.42±3.02
71-80	96	17.43±0.55	13.44±0.51	12.28±0.51	4.11±0.20	77.19±3.61
>80	20	17.20±0.89	12.84±0.72	12.26±0.39	4.21±0.12	74.65±2.14
One way ANOVA test	F-value	2.742	10.729	5.440	5.683	3.769
	P-value	0.008	<0.001	<0.001	<0.001	<0.001

The statistically significant association was observed among MCL (r= −0.722; P<0.001), MCB (r=0.616; P<0.001), IZL (r= −0.262; P<0.001), and OL (r= −0.191; P<0.001) with CI on applying the Pearson correlation (p<0.05); while statistically insignificant correlation was observed only age with CI (r= −0.018; P>0.05; Table 4).

**Table 4:** Pearson correlation *Correlation is significant at 0.01 level (two-tailed).

		Age	MCL	MCB	IZL	OL
CI	Pearson correlation	−0.018	−0.722^*^	0.616^*^	−0.262^*^	−0.191^*^
	Sig. (two-tailed)	0.568	0.000	0.000	0.000	0.000

The overall 47.2% study population shows the dolichocephalic (long head) in our study (Table 5).

**Table 5:** Distribution of the different skull shapes obtained in the study population

Skull Shapes	Frequency (n=1000)	Percentage (%)
Hyper dolichocephalic	4	0.4%
Dolichocephalic	472	47.2%
Mesocephalic	304	30.4%
Brachycephalic	220	22.0%

Dolichocephalic and brachycephalic type of skull was found more in 31-60 years age, and mesocephalic type of skull was more in ≤30 years age (Table 6).

**Table 6:** Age-wise distribution of study cases based on cephalic index and type of skull

CI group	Age group (years)			P-value
	≤30 (n=376)	31-60 (n=368)	>60 (n=256)	
Hyper dolichocephalic	0 (0.0%)	4 (1.1%)	0 (0.0%)	0.021
Dolichocephalic	168 (44.7%)	188 (51.1%)	116 (45.3%)	
Mesocephalic	124 (33.0%)	92 (25.0%)	88 (34.4%)	
Brachycephalic	84 (22.3%)	84 (22.8%)	52 (20.3%)	

**Figure 1:**
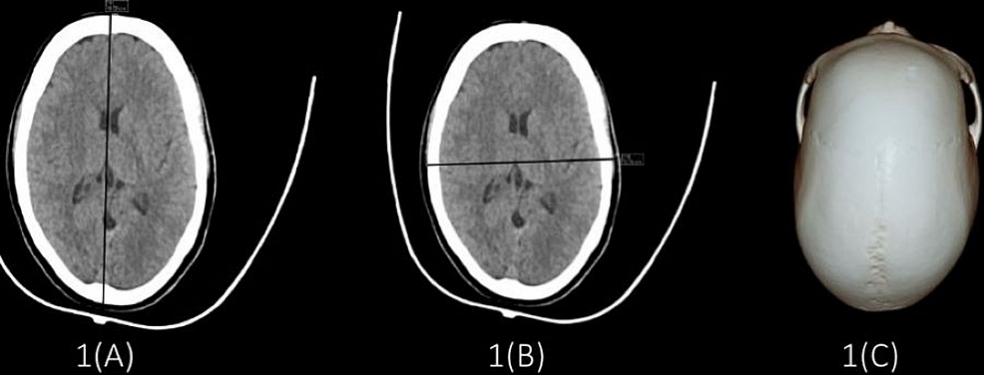
Images showing dolichocephalic skull (A) Maximum cranial length = 18.64 cm; (B) maximum cranial breadth = 13.21 cm; (C) cephalic index = 70.86.

**Figure 2:**
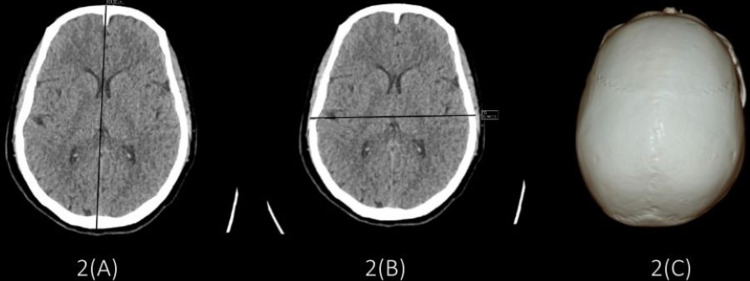
Images showing mesocephalic skull (A) Maximum cranial length = 17.63 cm; (B) maximum cranial breadth = 13.81 cm; (C) cephalic index = 78.33.

**Figure 3:**
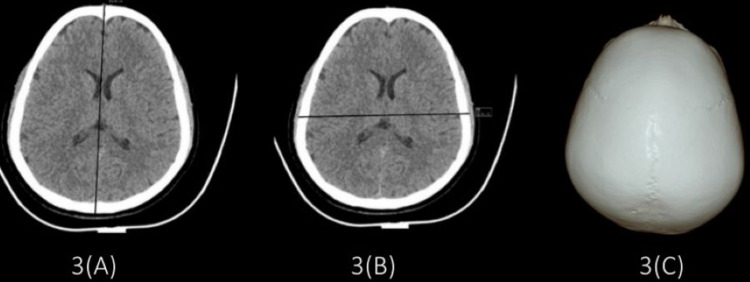
Images showing brachycephalic skull (A) Maximum cranial length = 16.52 cm; (B) maximum cranial breadth = 13.86 cm; (C) cephalic index = 83.89.

While dolichocephalic (Figure 1) and mesocephalic (Figure 2) type of skull was found more in male subjects, and brachycephalic (Figure 3) type of skull was more in female subjects (Table 7).

**Table 7:** Sex wise distribution of study cases based on cephalic index and type of skull

Cephalic index group	Sex		P value
	Male (n=540)	Female (n=460)	
Hyper dolichocephalic	4 (0.7%)	0 (0.0%)	<0.001
Dolichocephalic	316 (58.5%)	156 (33.9%)	
Mesocephalic	196 (36.3%)	108 (23.5%)	
Brachycephalic	24 (4.4%)	196 (42.6%)	

**Figure 4:**
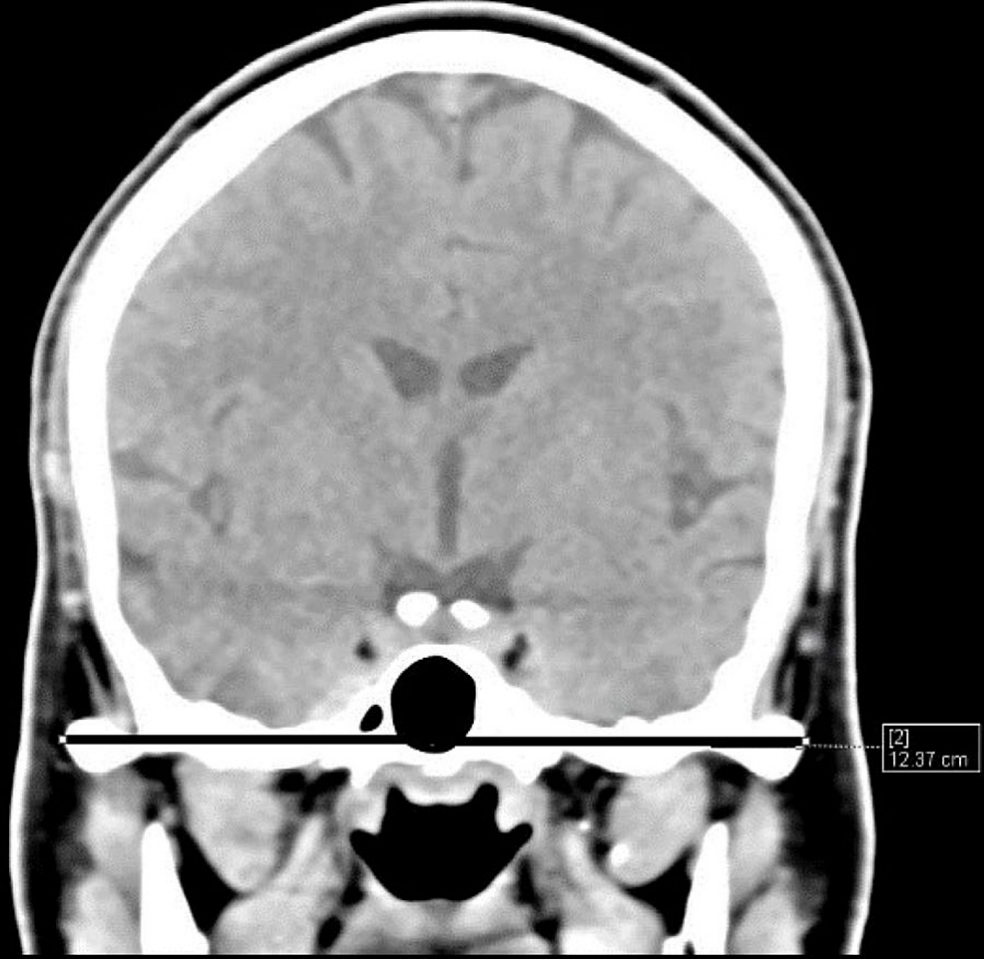
Interzygomatic length

**Figure 5:**
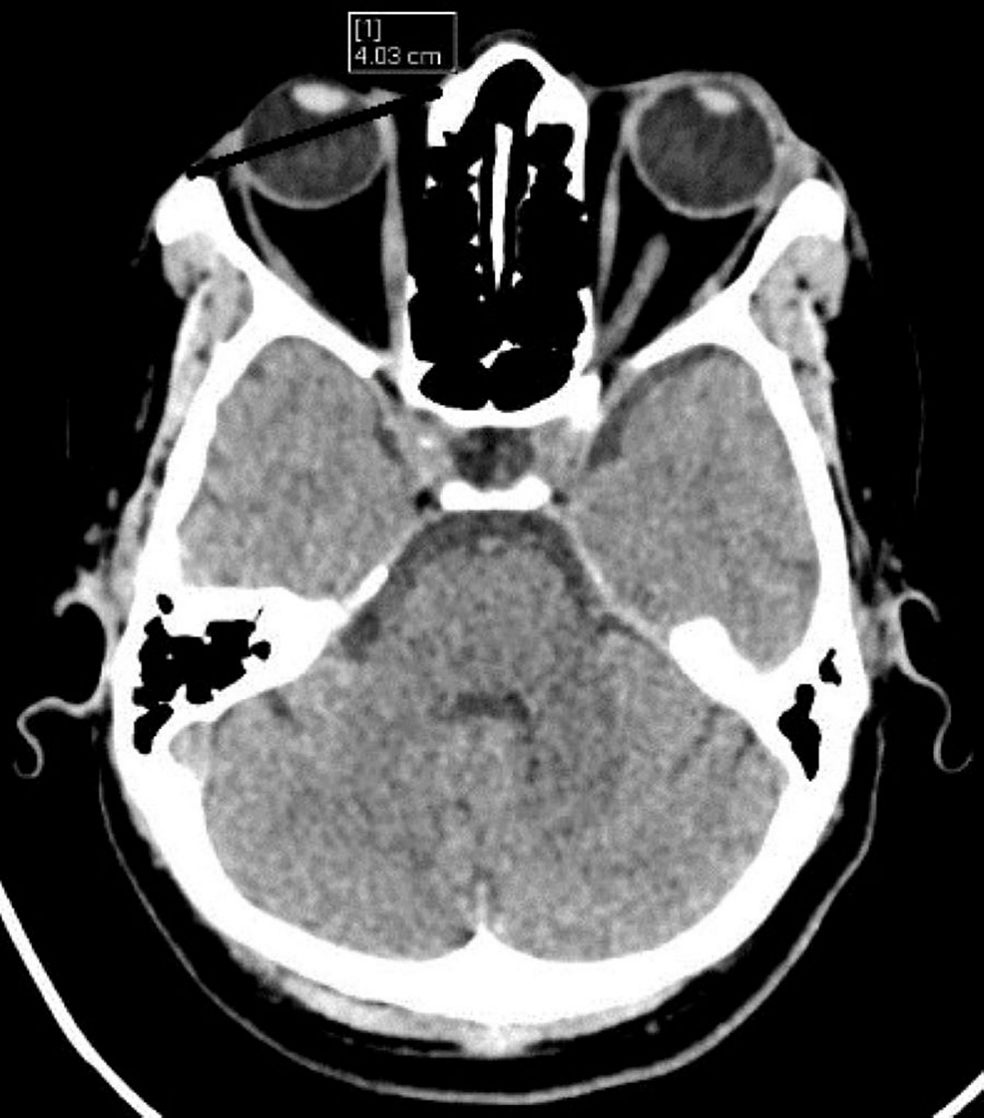
Orbital length

The distribution of MCL, MCB, IZL (Figure 4), OL (Figure 5), and mean CI in different age groups was statistically significant (p<0.05).

## Discussion

Craniofacial anthropometry is imperative in assessing facial trauma, identification of inherited deformity, defects, and diagnosing different diseases. It is essential to have indigenous data of these constraints since these values reflect the possibly different shapes of craniofacial development subsequent from ethnic, racial, and sexual differences [12].

There are different racial groups including Blacks, Asians, and Whites, and their differences are based on physical characteristics. On the other hand, there are serious genetic differences among different races. It is recognized that using a standard for the craniofacial structures is not suitable when building treatment and diagnostic planning decisions for patients from a diverse ethnic background.

The CI is an assessment scale to measure the skull size. CI rating is achieved by multiplying the maximum width of the head by 100 and dividing that whole number by the maximum length of the head. It has been planned as a discriminating parameter to identify variations in the growth of the head. Its evaluations when the calculated index is above ±2 SD from the mean. The analysis of the CI states another aspect of development and growth of the skull, likewise allows critical assessment of unusually small or large heads. Also, the CI indirectly expresses the cranial capacity that is used indirectly to reveal the volume of the brain and predict mental ability [13]. Additionally, no research has been carried out in open literature using CT scans to produce normative reference data. Therefore, this study aimed to utilize CT scans to quantify the differences in craniofacial morphology of North Indians with well-known published data for other populations and observe whether the differences change with age or gender.

The socio-demographic characteristics including the age and sex obtained from the present study (Table 1) were taken into consideration with respect to the cranial dimensions or parameters of the North India sampled population. On the gender distribution, results of our study showed no significant difference between females and males which corroborates similar work done by Ekizoglu et al. [14], on the evaluation of gender in the modern Turkish population through the cranial CT anthropometric parameters, and Paulinus et al. [15], also observed the same by cranial CT-anthropometric parameters over Nigerians residing in Calabar.

We performed a cross-sectional study on 1000 subjects. Akinbami [16] performed a cross-sectional study on the 700 subjects; Singh and Purkit [17] conducted a cross-sectional study on the 200 subjects while Paulinus et al. [15], did a retrospective and prospective study of over 200 subjects. The collection of data offered by our study probably covers the most extensive age range with a large number of subjects in each gender and age group. The results of our study indicated that adult males had higher values than adult females.

In this study, mainstream subjects belonged to the age group 21-30 years, i.e., 234 (23.4%) and the mean age of the total studied subjects was 43.43±20.71 years (range 6-95 years). In our study, male subjects 54.0% were dominant over females subjects (46.0%; Table 1). The MCL of subjects was 17.50±0.57 mm, mean MCB of heads was 13.40±0.39 mm, mean IZL was 12.17±0.49 mm, and OL was 4.10±0.19. The distribution of MCL, mean MCB, mean IZL, OL, and mean CI in different ages was statistically significant (p<0.05; Table 3).

We observed the mean CI of the male was 75.59±2.64. This finding was slightly lower than the studies conducted by Singh and Purkit [17] and Mishra et al. [18]. Singh and Purkit [17] reported the mean CI of males was 77.71±4.91, and Mishra et al. [18] reported 75.84 mean CI in males. Another Indian study from the Manipal region reported 77.92 mean CI in males [19] and Shah and Jadav [20] reported that CI's value was 80.42 in Gujrati males. The mean CI of females in this study was 77.94±3.30. This finding was also lower than the research done by Singh and Purkit [17] who reported that females' mean CI was 79.35 ±5.72. A study on resident Fars group with 85.0 in the north of Iran and Turkman group 82.8 in North of Iran [21], Shah and Jadhav [20], from India with 81.20, Manipal females with 80.85 [19]. But higher than the study in Tehran - the center of Iran with 75, Igbo (76.83) tribes community [22], Bayelsa State, Nigeria with 72.24 [23].

The mean CI of this study (combined population) was 76.67±3.18 which shows that the major head shapes among the North Indians are dolichocephalic and mesocephalic. Our finding was also lower than Singh and Purkit [17] study in India with 78.53 ± 5.38, Shah and Jadhav [20] study with 80.42, Mishra et al. [17], a study in Berelas of Central India was reported 77.79, study on Manipal population with 78.92±6.31 [19], a study in Port Harcourt, Nigeria with 79.80 [24]. But CI was more when compared to study in Tehran-Iran with 75 [21] and study by Eroje et al. [23], for Ogbia of Nigeria with 72.96. Hence, the present study's findings revealed that the sexual dimorphisms in cranial dimensions are pronounced best in mesocephalic form. The results of the present study agree with various other studies which compare anthropometric characteristics of females and males. Most of such writers have found the presence of sexual dimorphism in their respective studied samples. Oladipo et al. [25], on the facial measurements among chief ethnic groups in Nigeria where sexual dimorphism was observed in every ethnic group, studied with males having higher facial indices than females (p<0.05). Studies were done by Maina et al. [13] and Raji and Garba [26], who confirm comparable works on craniofacial classifications of normal newborn and morphological assessment of face and head shapes, all in a sampled north-eastern Nigerian population.

The age, MCL, MCB, IZL, OL, and CI on applying the Pearson correlation we find the statistically significant association with each other (p<0.054); but statistically insignificant correlation was observed only in age with CI and age with IZL (Table 4). Dolichocephalic and brachycephalic type of skull was found more in 31-60 years age, and mesocephalic type of skull was more in ≤30 years age (Table 5). While dolichocephalic and mesocephalic types of the skull were found more in male subjects, and brachycephalic type of skull was more in female subjects (Table 6).

In our study, the dominant head shape type was dolichocephalic (47.2%) followed by mesocephalic (30.4%). This was similar to a study performed by Ahmed et al. [27], who found 58.5% of the Indian population was dolichocephalic. Dominant head type in this study was not similar to other studies in Nigeria by Akinbami [16], the study in central India by Yagain et al. [19], the study of the Igbo tribe of Nigeria by Oladipo and Olotu [22], and the study in Port Harcourt, Nigeria by Fawehinmi and coworkers [23]. This shows that there is a tendency toward dolichocephalic. Comparing the previous records of the CI with current work proves the tendency towards "mesocephalic" that is a confirmation of continuous growth of brain more in lateral direction [20] (Table 8). Also, in tropical zones, the form of the head is longer (i.e., dolichocephalic), but in the temperate zones, the head type is round (i.e., mesocephalic or brachycephalic) [28]. Since India is in both temperate and tropical zones partly, the present classification depicts a tendency to dolichocephalic from brachycephalic.

**Table 8:** Cephalic Indices of different studies

Studies	Cephalic index (mean)
Akinbami [16]	76.86±2.54
Singh and Purkit [17]	78.53±5.38
Paulinus et al. [15]	75.95±4.09
The present study (2020)	76.67±3.18

Limitations of the study

Limited data on the craniofacial dimensions of the study population are a huge barrier in the precise estimation of the MCL and MCB. Also, this study is based on CT scans so there may be few variations in knowledge as no direct patient is involved.

Recommendations of the study

Further studies recommended on a gene variation basis are required to determine the specific genetic factors accountable for differences in cephalic indices among tribes, sexes, and races.

## Conclusions

The CI is a useful and indispensable tool used for assessing skull shape in adults as well as in children, especially for pre-and postoperative correction of skull deformations. We observed that dolichocephaly (47.2%) and mesocephaly (30.4%) are the dominant shapes of the head among the North Indians. Parameters of sexual dimorphism in the cranial dimensions have been examined and established in the Northern Indian population in the city Lucknow. Knowledge of cranial parameters and cranial index is essential in evaluating age, gender, and racial differences in clinical applications and forensic applications.
